# Chemotherapy increases caspase-cleaved cytokeratin 18 in the serum of breast cancer patients

**DOI:** 10.2478/v10019-011-0006-7

**Published:** 2011-03-15

**Authors:** Engin Ulukaya, Esra Karaagac, Ferda Ari, Arzu Y. Oral, Saduman B. Adim, Asuman H. Tokullugil, Türkkan Evrensel

**Affiliations:** 1 Medical School of Uludag University, Clinical Biochemistry Department, Bursa, Turkey; 2 Science and Art Faculty of Uludag University, Biology Department, Bursa, Turkey; 3 Medical School of Uludag University, Pathology Department, Bursa, Turkey; 4 Medical School of Uludag University, Medical Oncology Department, Bursa, Turkey

**Keywords:** apoptosis, chemotherapy, M30, response to chemotherapy, breast cancer

## Abstract

**Background:**

Apoptosis is thought to be induced by chemotherapy in cancer patients. Therefore, the measurement of its amplitude may be a useful tool to predict the effectiveness of cancer treatment sooner than conventional methods do.

**Patients and methods:**

In the study presented, apoptosis was assessed with an ELISA-based assay in which caspase-cleaved cytokeratin 18 (M30-antigen), a novel specific biomarker of apoptosis, is measured. Thirty seven patients with malignant (nonmetastatic and metastatic) breast cancer, 35 patients with benign breast disease, and 34 healthy subjects were studied. Cancer patients received neoadjuvant chemotherapy consisting of either fluorouracil, epirubicin, and cyclophosphamide (FEC) or epirubicin plus docetaxel (ED). Apoptosis was detected before chemotherapy, 24 and 48 h after chemotherapy in the malignant group.

**Results:**

It was found that the baseline apoptosis level in either malignant but nonmetastatic group or benign group was not statistically different from that in the control group (p>0.05). However, it was statistically significantly higher in the metastatic group than that in the control group (p<0.05). Following the drug application, M30-antigen levels significantly increased at 24 h (p<0.05). The baseline M30-antigen levels increased about 3-times in patients showing tumor regression.

**Conclusions:**

M30-antigen level is increased after chemotherapy and its measurement may help clinicians to predict the effectiveness of chemotherapy sooner in breast cancer cases although confirmative larger trials are needed.

## Introduction

Breast cancer is the leading cancer type which accounts for the highest mortality rate among woman cancers.^1^–^3^ Although new chemotherapeutic agents have been introduced into the market, the patient outcome is still not satisfactory.^3^ The improvement of the outcome may be achieved by an early prediction of the response to chemotherapy. For this aim, new biomarker(s), which provide information of the effectiveness of chemotherapy, are required.^4^ Apoptosis-related biomarkers may be of importance in this regard.

The mechanism by which chemotherapy kills the cancer cells is mainly the induction of the apoptotic pathway.^5^ Because the effects of anti-cancer drugs is based on the induction of apoptosis, *in vitro* evaluation of apoptosis has been used for testing the efficacy of anti-cancer agents.^6^–^8^ If apoptosis in serum can be measured by a biomarker which results from its induction, this may be of great importance for the clinicians to predict the response to chemotherapy they apply to their patients. It seems that there is such a biomarker which is found in the cytoskeleton.

Cytokeratin 18 (CK 18) is a member of cytoskeletal protein family which is present in epithelial cells.^9^ When apoptosis is induced, CK 18 is cleaved from aspartate amino acids localized at position 238 and 396. Monoclonal antibody M30 recognizes the neoepitope of CK 18 formed after cleavage by the caspases. This newly-formed neoepitope can be regarded as a selective biomarker of apoptosis.^10^,^11^ In fact, it was reported that the M30-antigen assay, which detects this neoepitope, reflects apoptosis accurately.^12^ It is also reported that M30-antigen is used as a marker for pharmocodynamic studies in cancer.^13^ Because deregulated apoptosis is a common feature of malignancies^14^, its assessment via circulating apoptotic markers have recently been made in some tumor types, such as gastrointestinal cancers.^15^ Recently, it was reported that serum M30-antigen levels may also be a prognostic marker in some tumor types.^16^,^17^ In another study, M30-antigen was reported to be associated with the survival in advanced gastric carcinoma patients.^18^

In addition to its being used as a prognostic marker in tumors, M30-antigen may provide important information regarding the response to therapy. Thus, it may be useful for the estimation of the efficacy of therapy.^19^ Kramer *et al*. presented that serum M30-antigen levels increased after docetaxel regimen in prostate cancer.^20^ Similarly, it was demonstrated that M30-antigen levels increased after chemotherapy in testicular cancer patients.^16^ Our group previously showed that M30-antigen levels increased as a response to therapy in breast cancer patients but we did not measure its levels in benign breast diseases and healthy subjects.^21^

Therefore, we investigated if M30-antigen is increased in breast cancer patients as well as in benign breast diseases in comparison with healthy subjects. We also measured its level after the application of chemotherapy in neoadjuvant setting. We found that chemotherapy leads to a significant increase in M30-antigen levels in serum of breast cancer patients. Thus, it may be used as a biomarker for the prediction of response to chemotherapy in breast cancer patients.

## Patients and methods

### Patient selection, treatment and assessment of clinical tumor response

The characteristics of the study participants are presented in Table 1. Patients with previously untreated, histological confirmed invasive breast cancer were eligible. The patient selection and eligibility criteria to be enrolled to the study were made according to the previous study performed by our group.^21^ Briefly, their performance status 2 by ECOG. Core needle biopsy was used for the histological confirmation of the tumor. The patients were treated with FEC or ED regimens: FEC regimen consisted of 5-fluorouracil (EBEWE Pharma, Austria) 500 mg/m^2^, epirubicin (EBEWE Pharma, Austria) 100 mg/m^2^, cyclophosphamide (BAXTER, Germany) 500 mg/m^2^ while ED regimen consisted of epirubicin 75 mg/m^2^ and docetaxel (EBEWE Pharma, Austria) 80 mg/m^2^. All drugs were administered on day 1, at every 21 days.

The response to treatment was assessed after the completion of four cycles of neoadjuvant chemotherapy by standard breast calipers and graded into: (a) clinical complete response (no tumor measurable); (b) clinical partial response (≥50% reduction in tumor size); (c) clinical stable disease (<50% reduction or an increase in tumor size of ≤50%); and (d) clinical progressive disease (>50% increase in tumor size or suspicious new lesion). According to the classification above, the complete and partial responses were defined as a regressive group. The other two groups were the stable group showing stable disease and the progressive group showing progression as given above. The informed consent was obtained from the participants and the local ethic committee approved the study.

### M30-antigen detection

The serum samples of malignant cases were collected prior to chemotherapy (baseline M30-antigen level), and 24 and 48 h after the treatment. Therefore, the acute (short term) effect of the therapy was actually assessed ignoring the long term effects. The sera of benign and healthy control subjects were collected at the time of admission only. The sera were stored at −80ºC until the assessment. An ELISA assay (a solid phase, two-site immunosorbent assay) was used to measure M30-antigen by using a commercial kit (M30-Apoptosense ELISA kit, Peviva, Sweden). Measurement was performed according to the instructions of the manufacturer. The absorbance was finally measured in a microplate reader (FlashScan, Jena, Germany) at 450 nm and the M30-antigen levels were estimated by the standard curve. The concentration of the M30-antigen was expressed as unit per liter (U/L).

### Histopathological evaluation

Tissue specimens were fixed in 10% buffered formalin (pH 7.4) and embedded in paraffin. 5 μm thick sections were cut and stained with hematoxylin and eosin. Estrogen (ER) and progesterone (PgR) receptor status were assessed by immunohistochemistry. All specimens were examined by an experienced pathologist who was unaware of the clinical data. Only 22 patients’ samples were accessible. The proportion of ER and PgR positive cells was determined as the percentage of invasive tumor cells. The threshold of 10% positivity was chosen as a cut-off value.

### Statistical analysis

The statistical analysis was performed using SPSS 13.0 (SPSS Inc., Chicago, IL, USA). All values are presented as mean (± standard deviation- S.D.) and median. In the case of the distribution of parameters did not show normal distribution, non-parametric statistics (Kruskal-Wallis and Mann Whitney-*U* tests) were used. Wilcoxon Rank Sum test was also used to compare two dependent samples represented by M30-antigen levels before and after chemotherapy. The relationship between M30-antigen levels and parameters were analyzed by Pearson Correlation. Statistical significance was assigned to *p*-values less than 0.05.

## Results

### Characteristics of the study groups

The characteristics of participants are given in Table 1. The healthy control group included 34 people, while the malignant (metastatic and non-metastatic) group and the benign group included 37 and 35 patients, respectively. Among the malignant group, most of them had invasive ductal carcinoma (n=29), while only 5 of them were metastatic breast cancer cases.

### Serum M30-antigen levels are elevated in metastatic breast cancer patients

The baseline level of serum M30-antigen was measured in control, benign and malignant groups. It was found that the mean M30-antigen levels in control, benign, nonmetastatic and metastatic group were 127 ± 46, 173 ± 224, 182 ± 336, and 333 ± 184, respectively. A statistically significant difference between the groups was observed (p<0.05; Kruskal-Wallis and Mann-Whitney U tests) (Table 2). The mean of the metastatic malignant group was significantly higher than either benign or control group (p<0.05). There was no significant difference between the control group and either the nonmetastatic malignant group or the benign group. The mean M30-antigen level of the metastatic group was significantly higher than that in the nonmetastatic group.

### M30-antigen level increases following chemotherapy

Eleven nonmetastatic breast cancer patients accepted to donate serum sample after chemotherapy. Blood samples were collected prior to chemotherapy and 24 and 48 h after the application of chemotherapy. The baseline, 24 h and 48 h after chemotherapy levels were 316 ± 564, 809 ± 1526, and 584 ± 874, respectively. The baseline M30-antigen levels increased more than 2-fold 24 h after chemotherapy (p<0.05) (Table 3). In addition, M30-antigen level at 48 h following chemotherapy was still higher than the baseline level but it was not statistically significant (p>0.05).

### Relationship between M30-antigen level and receptor status, stage, and routine tumor markers

It has been found that M30-antigen levels differ depending on the ER or PgR status. ER negative (183 ± 31) or PgR negative (235 ± 51) cases had higher M30-antigen levels compared to those in ER (153 ± 39) or PgR (148 ± 30) positive cases (p<0.05), respectively (Table 4).

M30-antigen levels differ depending on the stage (Table 5). Stage IV patients seem to have the highest levels. There was no significant difference between stage II and III but stage IV cases had significantly higher levels compared to those in either stage II or III.

The correlation between M30-antigen and the routine clinical chemistry parameters was analyzed. But there was no significant correlation between them (data not shown). In addition, there was no correlation between baseline M30-antigen levels and age (r=0.045, p=0.647). The levels of lactate dehydrogenase (LDH), alkaline phosphatase and platelet count were measured in patients with malignant breast cancer and healthy controls. There was no statistically significant difference between the groups (data not shown).

### Relationship between M30-antigen and tumor response to chemotherapy in neoadjuvant setting

Eleven malignant cases were classified into three groups according to their responses to chemotherapy: regressive group (n=5) consisting of clinical complete or partial responses, stable group (n=4), and progressive group (n=1). One patient’s response was not evaluated although post-chemotherapy M30-antigen level of that patient was available. As it is shown on Table 6, M30-antigen level of both the stable group and the progressive group did not significantly change after chemotherapy, while it sharply increased in the regressive group 24 h after chemotherapy. It increased about 3-fold (from 544 to 1633 U/L) in this group, implying the apoptosis-inducing effect of drugs applied. However, the differences were not statistically evaluated due to a low number of cases.

## Discussion

In the study presented, we measured the M30-antigen levels before and after chemotherapy to investigate its relation with response to treatment. We found that M30-antigen significantly increased following chemotherapy. This may give an idea of the effectiveness of chemotherapy applied in neoadjuvant setting. Neoadjuvant chemotherapy is increasingly being applied in the management of patients with large (≥ 3 cm) and locally advanced breast cancer. Although neoadjuvant chemotherapy may lead to similar disease-free and overall survival rates with those obtained with adjuvant chemotherapy, the response of breast cancer patients to neoadjuvant chemotherapy was found as the most important predictive factor for the survival.^22^–^24^ However, which patient would response to the neoadjuvant chemotherapy is still unpredictable. That is why we believe that the measurement of apoptosis, which is induced by anticancer drugs, may be of great importance in the prediction of response to therapy. This may be achieved by measuring the M30-antigen levels in serum and the clinicians may thus predict better the outcome of their patients by using this tool.

Death of tumor cells generates detectable protein products in the patient’s circulation, which may be used for cancer diagnostics and/or monitoring of therapy efficacy.^25^ Apoptosis is a form of regulated cell death that is characterized by specific structural changes, mediated by proteases of the caspase family.^26^ Caspase activity itself or the presence of specific degradation products can be used for the detection of tumor cell apoptosis. The M30 antibody detects a caspase-degraded product, CK18-Asp396 (also called M30-antigen), of the important cytoskeletal protein called cytokeratin 18 of epithelial cells. Cytokeratin 18 is expressed by most carcinomas, including those of breast, prostate, lung and colon.^11^

Treatment-induced changes in growth dynamics (apoptosis and proliferation) in breast cancer are essential to determine the response or resistance of tumors to chemotherapy. The early detection of chemosensitive tumor with the assessment of apoptosis or different techniques may facilitate the individualized-chemotherapy. It has previously been shown that circulating M30-antigen levels increased in patients with various cancer types and, furthermore, it increased during chemotherapy.^21^,^27^,^28^ For instance, the docetaxel treatment increased levels of M30-antigen in the serum of breast cancer patients, indicating apoptotic death of tumor cells, while the cyclophosphamide/ epirubicin/5-fluorouracil treatment led to a heterogenous response with regard to cell death mode.^29^ Our group previously reported that M30-antigen increased 4-fold after chemotherapy in lung cancer patients.^30^ In accordance with this finding, in this study presented, we observed that M30-antigen level was significantly increased 24 and 48 h after the chemotherapy in breast cancer patients. In fact, preclinical and clinical studies have shown that apoptosis significantly increases 1 to 3 days after chemotherapy administration.^31^–^33^

In the present study, we found that there was no statistically significant difference between the non-metastatic and the control group in terms of M30-antigen levels (p>0.05). In supporting this finding, there was no statistically significant difference between the malignant group (202 ± 84) and the control group (187 ± 58) in terms of baseline LDH level, which also represents cell death in serum. However, this may depend on the type of tumor. In the patients with disseminated testicular germ cell tumor, circulating M30-antigen levels were found to be correlated with classic prognostic markers including LDH probably reflecting tumor load.^16^

In contrast to inexistence of M30-antigen increase in the non-metastatic group compared to the control group, M30-antigen level was significantly higher in the metastatic group than that in the control group (p<0.05). This implies that the aggressiveness (metastatic ability) of tumor mass may have an impact on the serum level of M30-antigen. This increase may also be explained by the differentiation level of the tumor cells. It is highly possible that the stage or the total size of the tumor mass seems to affect the M30-antigen levels in serum. In fact, in the Olofsson’s study, there was a clear relationship between the tumor size and the M30-antigen levels.^29^ In this study, stage IV patients had much higher M30 antigen levels than those either stage II or III patients. Thereby, there must be a close link between apoptosis and both malignancy itself and the extension of malignancy. In agreement with this, we did not find any statistically significant increase in M30-antigen levels in the benign group, compared to the control group.

Several studies demonstrated that M30-antigen levels were higher in ER negative breast tumors than ER-positive tumors^21^,^28^, consistent with our results. Furthermore, we also found that M30-antigen levels were higher in PgR negative tumors, compared to PgR-positive ones. However, this needs to be confirmed by larger clinical studies. In fact, the weakness of our study was the low number of patients studied although the results were interesting.

## Conclusions

These findings indicate that serum M30-antigen is increased following FEC-based or ED-based chemotherapy. Thus, the measurement of its serum level may be a useful tool to predict the effectiveness of chemotherapy sooner in breast cancer patients. However, larger clinical studies are required to use it in the clinics routinely.

## Figures and Tables

**TABLE 1.** The characteristics of participants

**Characteristics**	**n**
Control	**34**
Mean age ±S.D. (47.2±10.6)	
Malignant group	**37**
Mean age ±S.D. (51.1 ± 12)	
Invasive ductal carcinoma	29
Invasive lobuler carcinoma	3
Metastatic breast cancer	5
Benign group	**35**
Mean age ±S.D. (40.6 ± 8.2)	
Fibrocystic	31
Fibroadenoma	4
Sex	All women
Stage	
I	4
II	17
III	11
IV	5
ER (+)	14
ER (−)	8
PR (+)	18
PR (−)	4

ER, estrogen receptor status; PR, progesterone receptor status

**TABLE 2.** M30-antigen levels in different groups

**M30-antigen**	**Control group (n = 34)**	**Benign group (n = 35)**	**Nonmetastatic group (n = 32)**	**Metastatic group (n = 5)**
Mean ± S.D.	127 ± 46	173 ± 224	182 ± 336	333 ± 184
(min-max)	71–340	68–1295	58–2010	159–607
Median	114	107	118	350
*p*-Value		>0.05*	>0.05**	<0.05***

* Comparison of control group and benign group, Mann-Whitney U test

** Comparison of control group and nonmetastatic group, Mann-Whitney U test

*** Comparison of control group and metastatic group, Mann-Whitney U test

**TABLE 3.** M30-antigen levels after chemotherapy (n=11)

**M30-antigen level (U/L)**	**Before chemotherapy (baseline level)**	**24 h after chemotherapy**	**48 h after chemotherapy**
Mean ±S.D	316 ± 564	809 ± 1526	584 ± 874
(min-max)	96–2010	98–4986	82–2586
Median	136	143	150
*p*-Value		<0.05*	>0.05**

* Comparison of M30-antigen levels before chemotherapy and those 24 h after chemotherapy, Wilcoxon Sign Rank test

** Comparison of M30-antigen levels before chemotherapy and those 48 h after chemotherapy, Wilcoxon Sign Rank test.

**TABLE 4.** The M30-antigen levels in ER(−), ER(+) and PgR(−), PgR(+) groups

**M30-antigen level (U/L)**	**ER (+)** **n=14**	**ER (−)** **n=8**	**PgR (+)** **n=18**	**PgR (−)** **n=4**
Mean ±S.D	153 ± 39	183 ± 31	148 ± 30	235 ± 51
(min.-max.)	58–607	86–383	58–607	146–383
*p*-Value	<0.05*		<0.05**		

Only 22 patients’ data were obtained for the evaluation of ER and PgR status.

* Comparison of ER(−) group and ER(+) group, Mann-Whitney U test

** Comparison of PgR(−) group and PgR(+) group, Mann-Whitney U test

**TABLE 5.** Comparison of the M30-antigen levels according to the stage of disease

**M30-antigen level (U/L)**	**Stage II (n=17)**	**Stage III (n=11)**	**Stage IV (n=5)**
Mean ±S.D	242 ± 110	117 ± 15	333 ± 184
(min-max)	72–2010	59–211	159–607
Median	131	113	351
*p*-Value	p<0.05*	p<0.05**	

* Comparison of the M30-antigen levels between stage 2 and 4, Mann-Whitney U test

** Comparison of the M30-antigen levels between stage 3 and 4, Mann-Whitney U test

**TABLE 6.** Classification into the responses to chemotherapy in the neoadjuvant setting and their M30-antigen values (U/L, ± SS)

	**Stable group, (n=4)**	**Regressive group, (n=5)**	**Progressive group, (n=1)**
Before Chemotherapy	137 ± 46	544 ± 820	110
24 h after Chemotherapy	115 ± 11	1633 ± 407	143
48 h after Chemotherapy	116 ± 23	1076 ± 1150	502

The statistical evaluation was not performed due to the low number of cases.

## References

[1] Benson JR, Jatoi I, Keisch M, Esteva FJ, Makris A, Jordan VC (2009). Early breast cancer. Lancet.

[2] Plesnicar A, Golicnik M, Fazarinc IK, Kralj B, Kovac V, Plesnicar BK (2010). Attitudes of midwifery students towards teaching breast-self examination. Radiol Oncol.

[3] Ovcaricek T, Frkovic SG, Matos E, Mozina B, Borstnar S (2011). Triple negative breast cancer – prognostic factors and survival. Radiol Oncol.

[4] Strojan P (2008). Cysteine cathepsins and stefins in head and neck cancer: an update of clinical studies. Radiol Oncol.

[5] Hickman JA, Beere HM, Wood AC, Waters CM, Parmar R (1992). Mechanisms of cytotoxicity caused by antitumour drugs. Toxicol Lett.

[6] Ohmori T, Podack ER, Nishio K, Takahashi M, Miyahara Y, Takeda Y (1993). Apoptosis of lung cancer cells caused by some anti-cancer agents (MMC, CPT-11, ADM) is inhibited by bcl-2. Biochem Biophys Res Commun.

[7] Walton MI, Whysong D, O’Connor PM, Hockenbery D, Korsmeyer SJ, Kohn KW (1993). Constitutive expression of human Bcl-2 modulates nitrogen mustard and camptothecin induced apoptosis. Cancer Res.

[8] Hagg M, Bivén K, Ueno T, Rydlander L, Björklund P, Wiman KG (2002). A novel high-through-put assay for screening of pro-apoptotic drugs. Invest New Drugs.

[9] Linder S (2007). Cytokeratin markers come of age. Tumour Biol.

[10] Ueno T, Toi M, Linder S (2005). Detection of epithelial cell death in the body by cytokeratin 18 measurement. Biomed Pharmacother.

[11] Leers MP, Kölgen W, Björklund V, Bergman T, Tribbick G, Persson B (1999). Immunocytochemical detection and mapping of a cytokeratin 18 neoepitope exposed during early apoptosis. J Pathol.

[12] Zhang L, Kavanagh BD, Thorburn AM, Camidge DR (2010). Preclinical and clinical estimates of the basal apoptotic rate of a cancer predict the amount of apoptosis induced by subsequent proapoptotic stimuli. Clin Cancer Res.

[13] Linder S, Olofsson MH, Herrmann R, Ulukaya E (2010). Utilization of cytokeratin-based biomarkers for pharmacodynamic studies. Expert Rev Mol Diagn.

[14] Holdenrieder S, Stieber P (2010). Circulating apoptotic markers in the management of non-small cell lung cancer. Cancer Biomarkers.

[15] Brandt D, Volkmann X, Anstätt M, Länger F, Manns MP, Schulze-Osthoff K (2010). Serum biomarkers of cell death for monitoring therapy response of gastrointestinal carcinomas. Eur J Cancer.

[16] de Haas EC, di Pietro A, Simpson KL, Meijer C, Suurmeijer AJ, Lancashire LJ (2008). Clinical evaluation of M30 and M65 ELISA cell death assays as circulating biomarkers in a drug-sensitive tumor, testicular cancer. Neoplasia.

[17] Wu YX, Wang JH, Wang H, Yang XY (2003). Study on expression of Ki-67, early apoptotic protein M30 in endometrial carcinoma and their correlation with prognosis. Zhonghua Bing Li Xue Za Zhi.

[18] Yaman E, Coskun U, Sancak B, Buyukberber S, Ozturk B, Benekli M (2010). Serum M30 levels are associated with survival in advanced gastric carcinoma patients. Int Immunopharmacol.

[19] Beachy SH, Repasky EA (2008). Using extracellular biomarkers for monitoring efficacy of therapeutics in cancer patients: an update. Cancer Immunol Immunother.

[20] Kramer G, Erdal H, Mertens HJ, Nap M, Mauermann J, Steiner G (2004). Differentiation between cell death modes using measurements of different soluble forms of extracellular cytokeratin 18. Cancer Res.

[21] Demiray M, Ulukaya EE, Arslan M, Gokgoz S, Saraydaroglu O, Ercan I (2006). Response to neoadjuvant chemotherapy in breast cancer could be predictable by measuring a novel serum apoptosis product, caspase-cleaved cytokeratin 18: a prospective pilot study. Cancer Invest.

[22] Fisher B, Bryant J, Wolmark N, Mamounas E, Brown A, Fisher ER (1998). Effect of preoperative chemotherapy on the outcome of women with operable breast cancer. J Clin Oncol.

[23] Bonadonna G, Valagussa P, Brambilla C, Ferrari L, Moliterni A, Terenziani M (1998). Primary chemotherapy in operable breast cancer: eight-year experience at the Milan Cancer Institute. J Clin Oncol.

[24] Scholl SM, Beuzeboc P, Harris AL, Pierga JY, Asselain B, Palangié T (1998). Is primary chemotherapy useful for all patients with primary invasive breast cancer? Recent results. Cancer Res.

[25] Holdenrieder S, Stieber P (2004). Apoptotic markers in cancer. Clin Biochem.

[26] Degterev A, Yuan J (2008). Expansion and evolution of cell death programmes. Nat Rev Mol Cell Biol.

[27] Ozturk B, Coskun U, Sancak B, Yaman E, Buyukberber S, Benekli M (2009). Elevated serum levels of M30 and M65 in patients with locally advanced head and neck tumors. Int Immunopharmacol.

[28] Ueno T, Toi M, Bivén K, Bando H, Ogawa T, Linder S (2003). Measurement of an apoptotic product in the sera of breast cancer patients. Eur J Cancer.

[29] Olofsson MH, Ueno T, Pan Y, Xu R, Cai F, van der Kuip H (2007). Cytokeratin-18 is a useful serum biomarker for early determination of response of breast carcinomas to chemotherapy. Clin Cancer Res.

[30] Ulukaya E, Yilmaztepe A, Akgoz S, Linder S, Karadag M (2007). The levels of caspase cleaved cytokeratin 18 are elevated in serum from patients with lung cancer and helpful to predict the survival. Lung Cancer.

[31] Meyn RE, Stephens LC, Hunter NR, Milas L (1995). Apoptosis in murine tumors treated with chemotherapy agents. Anticancer Drugs.

[32] Ellis PA, Smith IE, McCarthy K, Detre S, Salter J, Dowsett M (1997). Preoperative chemotherapy induces apoptosis in early breast cancer. Lancet.

[33] Green AM, Steinmetz ND (2002). Monitoring apoptosis in real time. Cancer J.

